# Effects of After-School Volleyball Program on Body Composition in Overweight Adolescent Girls

**DOI:** 10.3390/children9010021

**Published:** 2021-12-29

**Authors:** Nebojša Trajković, Anja Lazić, Drena Trkulja-Petković, Valentin Barišić, Vladan Milić, Siniša Nikolić, Goran Sporiš

**Affiliations:** 1 Faculty of Sport and Physical Education, University of Niš, 180000 Nis, Serbia; anja.lazic96@hotmail.com; 2 Faculty of Kinesiology, University of Zagreb, 10110 Zagreb, Croatia; drena.trkulja-petkovic@kif.unizg.hr (D.T.-P.); valentin.barisic@kif.unizg.hr (V.B.); sporis.79@gmail.com (G.S.); 3 Study Program of Sport and Physical Education, Department of Biomedical Sciences, State University of Novi Pazar, 36300 Novi Pazar, Serbia; vladan.milic@yahoo.com; 4 Department of Physical Medicine and Rehabilitation “Dr Miroslav Zotović”, 78000 Banja Luka, Bosnia and Herzegovina; sinisamnikolicbl@gmail.com; 5 Faculty of Sport and Physical Education, University of Novi Sad, 21000 Novi Sad, Serbia

**Keywords:** girls, vigorous intensity, body weight status, ball game

## Abstract

The current study aimed to investigate the effects of an after-school volleyball program on body composition in overweight adolescent girls. Forty-two girls were randomly divided into a volleyball group (VG) (*n* = 22 age: 15.6 ± 0.5 years) and control group (CG) (*n* = 20; age: 15.5 ± 0.7 years). Both groups continued with their usual physical education activities, while VG was included as small-sided games, two times a week, after school, on modified volleyball courts. Body mass, body mass index (BMI), body fat in kg, body fat percentage, and muscle mass were analyzed by a bioelectrical impedance method. There was a significant interaction of group (VG vs. CG) × time (pre-vs. post) for weight [F_1, 40_ = 7.933; *p* = 0.004] and BMI [F_1, 40_ = 5.764; *p* = 0.015]. Additionally, a significant main effect of time was found for body fat (kg) [F_1, 40_ = 17.650; *p* < 0.001] and body fat (%) [F_1, 40_ = 18.721; *p* < 0.001]. The results of the current study show that a twelve-week after-school volleyball program, including two sessions a week, can improve body composition in overweight adolescent girls.

## 1. Introduction

Nowadays, in a time of accelerated technological development, obesity in children and adolescents is taking on the character of an epidemic. The number of overweight and obese individuals worldwide has increased from 30 years ago, and, now, almost a third of the world population is defined as obese or overweight [1]. Concerningly, the majority of adolescents do not meet physical activity recommendations despite well-documented health benefits [2]. Although adolescence is a period when long-term healthy eating and exercise habits are formed, more than 340 million young people in the world were overweight or obese in 2016, and this negative trend continues to progress [3]. Accordingly, this age has been recognized as the period that may play a crucial role in the buildout and persistence of obesity and for leading to negative consequences during adulthood. Therefore, an effective approach to engage with youth and ameliorate their body composition status is of great importance. Children and adolescents spend most of their day in school. Therefore, the school setting has been recognized as a potentially effective environment for different exercise interventions [4]. Even though school interventions are effective and practical [5], previous studies [6,7] claimed that most adolescents are inactive during physical education classes and that the intensity of exercise is low. In accordance with all the above, there is a need for additional or different programs.

A previous review concluded that high-intensity interval training (HIIT) could be an effective method in accordance with the short duration of the activity for improving health-related indicators in adolescents [8]. However, there is no clear consensus on the positive effects of HIIT on body composition. Volleyball is considered to be one of the most played team sports worldwide. It is a sport that attracts all structures of the population; in particular, it involves no direct contact with the opponent but requires speed of movement and thinking. Volleyball, as an intermittent and complex activity where high-intensity elements are interrupted by a short period of low-intensity activities [9], could be included in work with obese female adolescents. Taking into account the previous statements that the type, configuration, and popularity of physical activity are required in both childhood and adolescent periods [10], volleyball appears to be an appropriate solution. Additionally, volleyball is one of the most popular physical activities in the aforementioned population [11]. Moreover, the total intensity of movements, according to the ratio of working metabolic rate (MET), can be measured at 6 METs in volleyball [12], which is considered to be vigorous [13]. Consequently, it is important to determine whether or not including volleyball as the only additional physical activity in PE classes can lead to an adjustment in body composition parameters and thus provide considerable health benefits.

Considering that volleyball has its popularity predominantly among the female population, it was of great interest to determine how it affects the body composition of female adolescents. To the best of the authors’ knowledge, to date, no study has focused on investigating the effects of small-sided volleyball games on body composition in overweight female adolescents.

Therefore, this study aimed to investigate the effects of a volleyball after-school program on body composition in overweight adolescent girls. We hypothesized that the exercise intervention would cause greater changes in body composition in overweight adolescent girls compared to physical education classes only.

## 2. Materials and Methods

### 2.1. Participants

This was a randomized study comparing an after-school volleyball program with traditional physical education classes in overweight female adolescents. Forty-two adolescent female participants from various classes of schools across Southern Serbia were included in the study. The participants were randomly divided into a volleyball group (VG) (*n* = 20; height: 158.97 ± 5.39; age: 15.6 ± 0.5 years) and control group (CG) (*n* = 22; height: 159.96 ± 10.71; age: 15.5 ± 0.7 years). Both groups continued with their usual physical education activities. During the volleyball program, there were no time-loss injuries.

The participants did not have any known medical problems. To be included, the participants had to be aged between 14 and 16 years, present a body mass index (BMI) ranging from the 85th to 95th percentile according to gender and age [14], be free of any medications that could impact the results, and not be involved in any systematic physical activities at the time of study or at least six months before the study (besides regular physical education classes at school, lasting up to 90 min/week).

All participants and parents/guardians were fully informed of the possible risks associated with the experimental procedures and gave their consent to participate in the study. Moreover, the participants and parents/guardians were informed that they could leave the experimental program at any time. The study protocol was approved by the ethics committee of the Faculty of Sport and Physical Education in Niš (Ref. No. 20/2020) and carried out in accordance with the Declaration of Helsinki.

### 2.2. Procedures

Measurement of body composition of all participants at the initial and final measurement was performed in the same school hall. The measurements were performed early in the morning, before 10 a.m., on both occasions. The testing was conducted by the same researchers on the initial and final measurement and in identical order.

Participants were measured in minimal clothing and barefoot for height and weight. Body height was assessed to the nearest 0.1 cm using a stadiometer (SECA Instruments Ltd., Hamburg, Germany). Body mass, BMI, body fat %, body fat in kg, and muscle mass were assessed via a bioelectrical impedance method using a standardized body composition analyzer, the InBody230 (InBody Co., Ltd., Cerritos, CA, USA). BMI was calculated using the following formula: BMI = (Weight in kilograms)/(Height in meters) squared. The InBody230 is a reliable device for women and men with high ICC for BF% (≥0.98) and has a low standard error of measurement [15]. Before the measurements, the participants were advised to excrete and refrain from drinking excessive amounts of liquids and not to deviate from their typical breakfast habits.

### 2.3. Exercise Intervention

It is widely accepted that children and adolescents should be engaged in strenuous or moderate physical activity for at least 60 min a day, which represents 420 min/week [16]. We included volleyball activities, played as small-sided games, two times a week after school. The experimental program lasted for 12 weeks. Volleyball was played on a court (4.5 to 6 m × 9 to 12 m), and the number of players was modified from 3 vs. 3 to 5 vs. 5 players. Each session started with a warm-up lasting 10–12 min. Moderate-intensity jogging was performed at the beginning of the warm-up session (3 min), along with dynamic stretching (4 min), and a specific volleyball warm-up with the ball (5 min). The main part of the session consisted of 40 min of volleyball. The session ended with a 5 min cool-down period. The intensity of training was monitored using the rate of perceived exertion (RPE) with a 10-point scale, collected during the training period. The CG undertook their regular PE classes involving team ball games (handball, basketball, or soccer) and individual sporting activities most common in many countries (track and field, gymnastics, or table tennis). In both groups, volleyball was excluded from regular PE classes. Both groups were not engaged in other organized physical activities besides organized volleyball activities and PE classes. The participants who had 70 > percent of attendance during the 12 weeks were taken for further analysis. There was no sample dropout during the experimental program and all participants were included in further analysis.

### 2.4. Statistical Analysis

Statistical analysis was performed with SPSS statistical program version 22 (SPSS Inc., Chicago, IL, USA). To test the normality, we performed the Kolmogorov–Smirnov test, which showed that the data were normally distributed (*p* > 0.05). Furthermore, Levene’s tests were determined for each dependent variable. The effects of the experimental treatment were analyzed using a two-way analysis of variance (ANOVA) in which the main and interaction effects (group × time) were calculated. Moreover, we used the Cohen’s d effect size (ES) with the use of the following criteria: trivial 0.0–0.2; small 0.2–0.6; moderate 0.6–1.2; large 1.2–2.0; and very large > 2.0 [17]. In addition to the statistical significance, which was set at *p* < 0.05, a partial eta squared (η^2^) was used for the differences between groups using 0.01, 0.06, and 0.14, determined as a small effect, medium effect, and a large effect [18].

## 3. Results

### 3.1. Adherence to the Exercise Program

The average attendance for the experimental program was 92%. The main reasons for skipping the exercise sessions were menstrual cycle, cold, and fatigue.

### 3.2. Body Composition

The results for body composition are presented in Table 1. There was a significant interaction of group (VG vs. CG) × time (pre- vs. post) found for weight [F_1, 40_ = 7.933; *p* = 0.004] and BMI [F_1, 40_ = 5.764; *p* = 0.015]. Additionally, a significant main effect of time was found for body fat (kg) [F_1, 40_ = 17.650; *p* < 0.001] and body fat (%) [F_1, 40_ = 18.721; *p* < 0.001].

## 4. Discussion

The number of overweight and obese children worldwide has risen alarmingly in the last twenty years [19], partly due to insufficient physical activity and unhealthy eating habits. The school environment is the setting where adolescents usually spend most of their day. Therefore, the current study aimed to investigate the effects of a 12-week school-based volleyball intervention on body composition in overweight female adolescents.

The most important results of the current study were the decrease in body mass and BMI after a relatively short volleyball program. Furthermore, the volleyball program over 12 weeks reduced body fat over time but showed no difference between the groups.

Obesity during childhood and adolescence, low cardiorespiratory fitness, and decreased physical activity have a strong association with various cardiovascular diseases [20]. Strategies to improve body composition status include diet, exercise interventions, or both. A recent study indicated that there is a general trend towards larger changes in body composition following HIIT protocols [8]. Volleyball is a sport that belongs to high-intensity activities [21], and it is predominantly popular in the female population; since it involves less high-intensity activity than other sports [22,23,24], it could be a suitable and effective activity for both obese and overweight adolescents. The current results, although among overweight adolescents, showed that the volleyball after-school program was more effective than those of the CG, with a 6.9% reduction in body mass after the experimental treatment (medium ES). Considering the baseline differences between CG and VG at the initial assessment, after the experimental program, there was a decrease in body mass index (BMI) in both groups. The reduction was greater in the VG (−5.3%) compared to CG (−0.8%). However, BMI is not the most adequate parameter to describe real differences in body composition, because a change in BMI does not mean that there has been an increase in muscle mass and a reduction in fat mass at the same time [25].

Although the current results showed a higher percentage decrease in body fat (4.3% in the VG and 2.1% in the CG group) and body fat in kg (5.5% in the VG and 1.5% in the CG) in the VG compared to the CG, the changes were not statistically significant (*p* > 0.05). A possible reason for the non-significant changes could be that both the CG and VG were exposed to physical activity for a period of 12 weeks. However, even the lowest level of increased physical activity leads to positive changes in the overweight and obese population. Moreover, adolescence is a period related to increased hormonal work and increased biological growth and development [26], where participants can be exposed to a longer duration of physical activity [27]. Another reason may be that, during the experimental program, the participants did not have any kind of educational content or control of nutrient intake that would affect the outcome [28].

It should be acknowledged that the study had some limitations. Firstly, physical activity was not measured during and outside school, which could be recognized as a limitation. Secondly, the small sample size of participants could also be considered as a limitation, particularly given that only overweight girls were included in the study. Third, dietary habits and total calorie intake were not controlled. However, they were instructed to maintain their usual calorie intake. Finally, after the experimental program and the final measurement, there was no further monitoring of the participants in the parameters of body composition, in order to determine whether there was a return of values to the initial level. Nevertheless, this study justifies the use of volleyball in the planning of physical education classes and after-school activities, showing that playing volleyball could significantly improve the body composition of overweight girls.

## 5. Conclusions

Overall, our findings show that an after-school volleyball program over twelve weeks is suitable for improving body composition in overweight adolescent girls. This study also shows that playing volleyball, added to regular PE classes, has the potential to reduce or even compensate for the limitations of the exercise program in a regular class in a relatively short time.

## Figures and Tables

**Table 1:** Results for body composition; results are presented as mean ± standard deviation (SD).

Variable	Group	Pretest	Posttest	ES	% Change	*p*-Value, η^2^_p_
Body mass (kg)						
	VG	62.55 ± 5.18	58.20 ± 5.58	−0.54	−6.9%	Group: *p* = 0.596, η^2^_p_: 0.006 Time: *p* < 0.001, η^2^_p_: 0.693 Interaction: *p* = 0.004, η^2^_p_: 0.164
	CG	61.55 ± 5.29	60.51 ± 5.74	−0.18	−1.7%	
BMI (kg/m^2^)						
	VG	24.52 ± 1.37	23.22 ± 1.46	−0.98	−5.3%	Group: *p* = 0.458, η^2^_p_: 0.013 Time: *p* < 0.001, η^2^_p_: 0.697 Interaction: *p* = 0.015, η^2^_p_: 0.211
	CG	25.19 ± 1.20	24.99 ± 1.66	−0.21	−0.8%	
Muscle mass (kg)						
	VG	20.42 ± 3.46	20.66 ± 3.60	+0.07	+1.2%	Group: *p* = 0.425, η^2^_p_: 0.014 Time: *p* = 0.393, η^2^_p_: 0.016 Interaction: *p* = 0.393, η^2^_p_: 0.016
	CG	19.68 ± 3.79	19.71 ± 3.69	0.0	0.1%	
Body fat (kg)						
	VG	13.94 ± 4.44	12.79 ± 4.59	−0.16	−5.5%	Group: *p* = 0.885, η^2^_p_: 0.000 Time: *p* < 0.001, η^2^_p_: 0.280 Interaction: *p* = 0.090, η^2^_p_: 0.065
	CG	13.35 ± 4.49	13.15 ± 4.24	−0.05	−1.5%	
Body fat (%)						
	VG	25.74 ± 5.27	24.64 ± 5.14	−0.14	−4.3%	Group: *p* = 0.425, η^2^_p_: 0.026 Time: *p* < 0.001, η^2^_p_: 0.312 Interaction: *p* = 0.124, η^2^_p_: 0.064
	CG	24.35 ± 5.32	23.83 ± 5.20	−0.10	−2.1%	

Abbreviations: VG, volleyball group; CG, control group; BMI, body mass index; ES, Cohen’s d effect size.

## Data Availability

Not applicable.
